# The complete chloroplast genome of *Cynoglossum amabile* Stapf & J. R. Drumm., 1906 (Boraginaceae), a traditional Chinese herbal medicine

**DOI:** 10.1080/23802359.2022.2160219

**Published:** 2023-01-02

**Authors:** Zhen-Ning Zhao, Xiao Yu

**Affiliations:** a School of Forestry, Southwest Forestry University, Kunming, China; b School of Landscape Architecture and Horticulture Sciences, Southwest Forestry University, Kunming, China

**Keywords:** Chloroplast genome, Boraginaceae, *Cynoglossum amabile*, phylogenetic tree

## Abstract

*Cynoglossum amabile* Stapf & J. R. Drumm., 1906 is a traditional Chinese herbal medicine from southwest China. To better determine its phylogenetic relatedness to other Boraginaceae species, the chloroplast (cp) genome of *C. amabile* was sequenced. The complete cp genome of *C. amabile* is 151,532 bp in length, containing a small single-copy (SSC) region with a length of 17,366 bp, a large single-copy (LSC) region with a length of 82,902 bp, and a pair of inverted repeats (IRs) regions each with a length of 25,632 bp. The overall GC content of the cp genome is 37.4%. The maximum-likelihood phylogenetic tree showed that *Bothriospermum zeylanicum* (J. Jacq.) Druce, 1917 was closely related to *C. amabile*.

## Introduction

**Figure 1.**
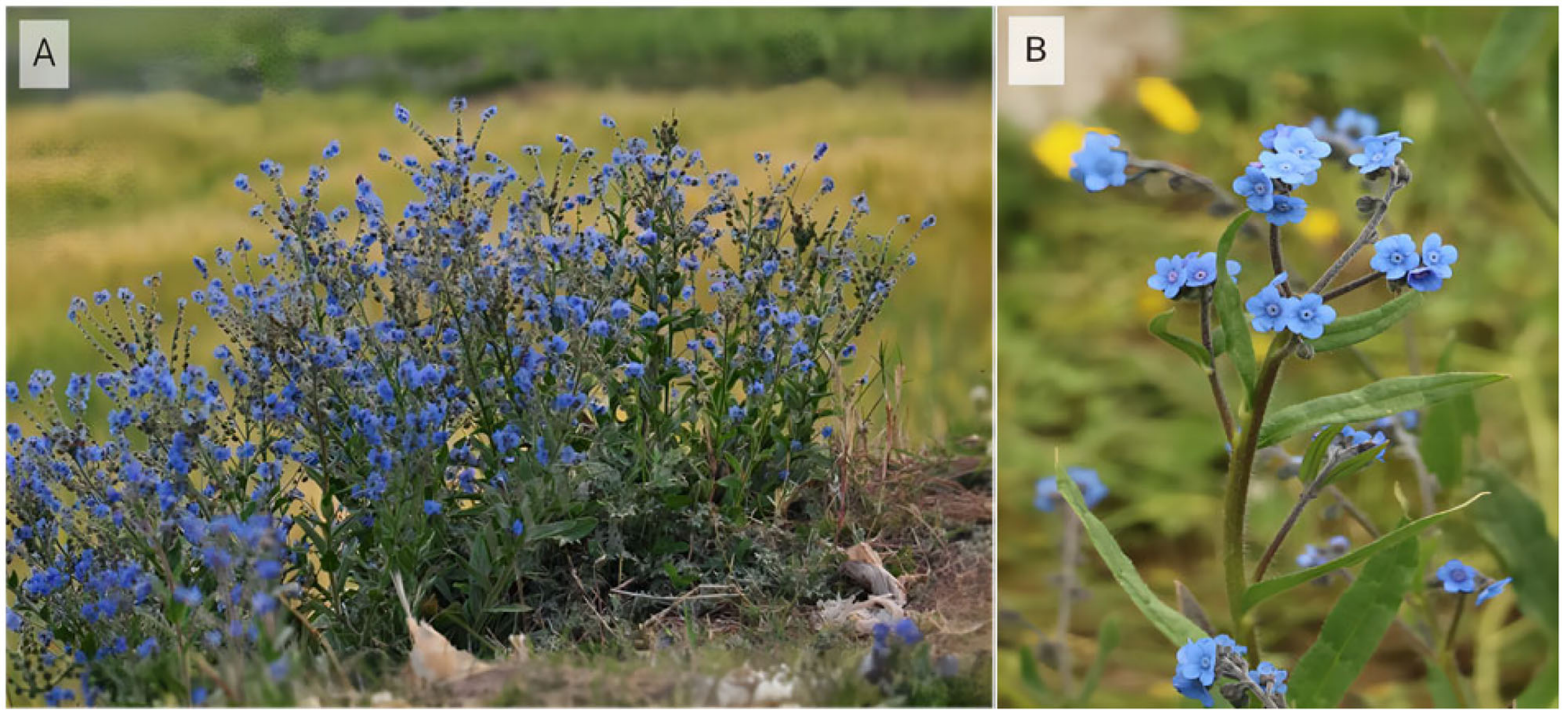
Plant morphological characteristics of *Cynoglossum amabile.* This species has densely spread pubescence. The photos of *Cynoglossum amabile* were taken by the authors in Yulong County, Yunnan Province, China (coordinates: 100°8′59.93″E, 26°46′8.02″N).

*Cynoglossum amabile* Stapf & J. R. Drumm., 1906 is a perennial herb within the family Boraginaceae, and is widely distributed in the mountainous areas of southwest China (Figure 1). It grows in hillside meadows, alpine shrubs, dry roads, or coniferous forest edges at an altitude of 1250–4565 meters (Zhang et al. 2014). This species is one of the most common ethnic medicinal materials in the producing area. Local people often use it to treat cough, vomiting, fractures, dysentery, and other diseases (Li and Zhao 2010). Studies have shown that *C. amabile* contains a variety of alkaloids, of which the main components are pyrrolizidine alkaloids with high clinical value. In addition, extracts of *C. amabile* were shown to have inhibitory effects on certain bacteria and fungi (Wang et al. 2014), indicating the plant’s high medicinal potential. To date, its chloroplast (cp) genome has yet to be characterized. cp genomes are valuable tools for determining species identity (Li et al. 2015), phylogenetic relationships, and germplasm diversity. Here, we reported the complete cp genome sequence of *C. amabile*, aiming to provide a useful resource for the phylogenetic studies of Boraginaceae.

## Materials and methods

### DNA extraction and sequencing

The fresh leaves of *C. amabile* were collected from Nanxi Village, Huangshan Town, Yulong County, Yunnan Province, China (coordinates: 100°8′59.93″E, 26°46′8.02″N; altitude: 3103 m). The collection of plant materials was in accordance with local regulations under the permission of local authorities. The voucher specimen (SWFU20210756MFY) was deposited at the herbarium of Southwest Forestry University, China (http://bbg.swfu.edu.cn/, Yu Xiao, email: yuxiao0215@gmail.com). The total genomic DNA was extracted from dried plant leaf specimens using the CTAB extraction method (Doyle and Doyle 1987). The paired-end sequencing reads were generated by the Illumina HiSeq 2500 sequencing platform (Illumina, San Diego, CA). After removing the poor-quality reads, 3.38 Gb clean data were obtained. The complete cp genome was assembled using NOVOPlasty with the following parameters: wordize = 102; base coverage = 171.44; and *k* = 75, 85, 95, 105, 115, and 127 (Dierckxsens et al. 2017). The complete cp genome of *C. amabile* was a typical quadripartite structure (Figs. S1 and S2). The cp genome was annotated in Geneious Prime ver. 2021.2.2 (Kearse et al. 2012) using *Trigonotis peduncularis* (MZ911745.1) as the reference sequence. The cp genome sequence of *C. amabile* has been submitted to GenBank under the accession number NC_061706. The cpgview (http://www.1kmpg.cn/cpgview) was used to draw the physical map of the cp genome.

### Simple sequence repeat analysis

The presence of SSR loci in the cp genome of *C. amabile* was analyzed using the online detection tool MISA-web (https://webblast.ipk-gater-sleben.de/misa/) (Beier et al. 2017). The minimum number of repeats was set to 10 for mononucleotides; five for dinucleotides; and four for trinucleotides, tetranucleotides, and pentanucleotides.

### Phylogenetic analysis

A phylogenetic tree was reconstructed to ascertain the phylogenetic relationship of *C. amabile* and 13 other species of Boraginaceae, with *Agastache rugosa* and *Ajuga forrestii* as outgroup taxa (Table S1). The MAFFT ver. 7 program (scoring matrix = 200, PAM *k* = 2, gap open penalty = 1.53, offset value = 0.123) was used for multiple alignment between the cp genome sequences of these 16 taxa, and the sequence differences were identified (Katoh and Standley 2013). Then, the alignment results were checked using Geneious Prime with the output file in the format of *.net. A maximum-likelihood (ML) tree was constructed with RAxML ver. 8.0.0 (m = GTR + GAMMA, bootstrap = 1000) (Stamatakis 2014) and was visualized with FigTree ver. 1.4.3 (http://tree.bio.ed.ac.uk/software/figtree/).

## Results and discussion

### Structural characteristics

**Figure 2.**
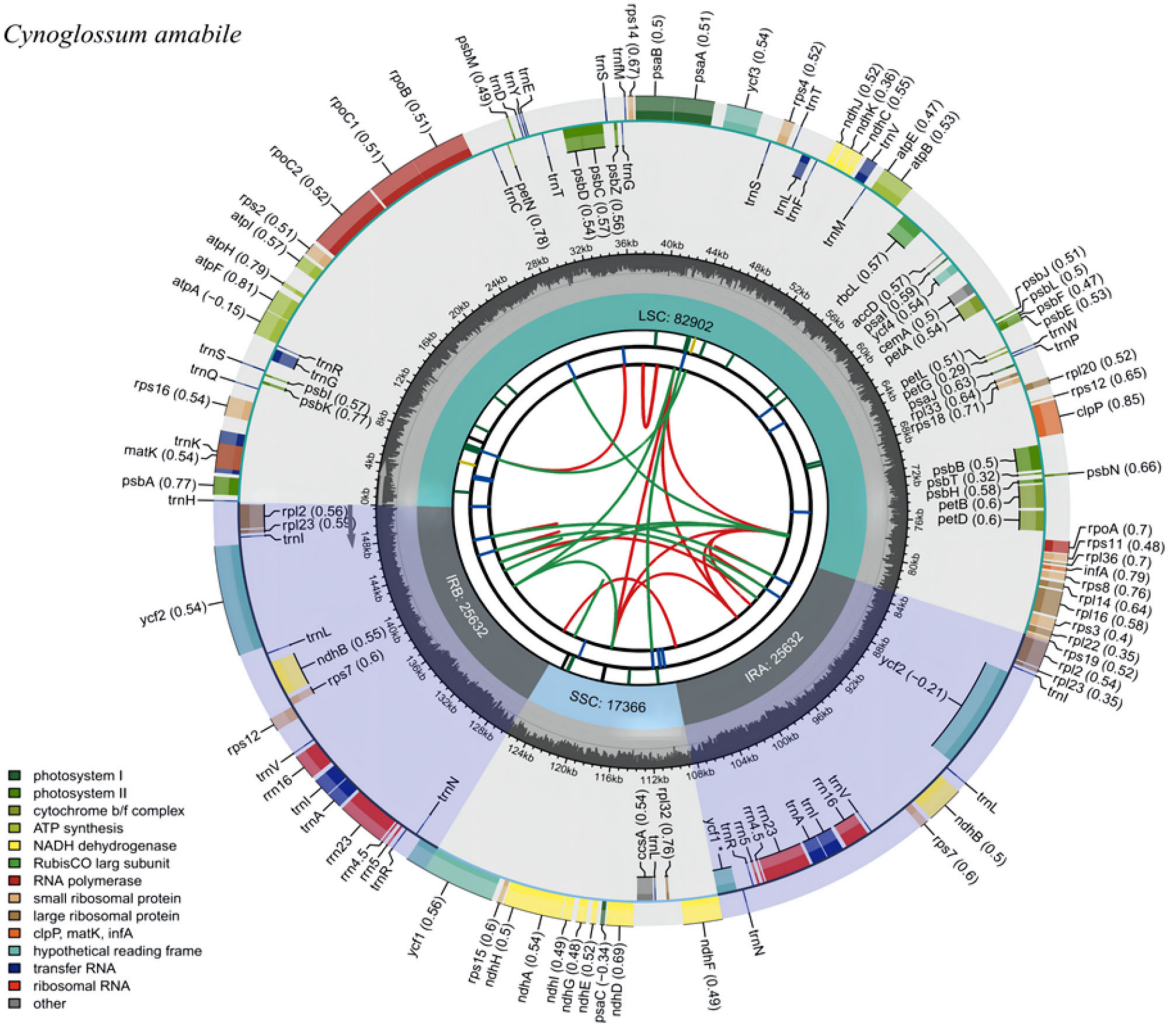
Gene map of *Cynoglossum amabile* chloroplast genome. The figure consists of several circles, and the information about each circle from the center to outward is as follows: the circle nearest to the center shows the forward and reverse repeats by red and green arcs, respectively. The second and third circles indicate the tandem repeats and microsatellite sequences by short bars, respectively. The fourth circle shows the position of the LSC, SSC, IRA, and IRB regions, respectively. The fifth circle shows the GC content. The outer circle indicates the gene functions. Different colors are used to show different functional categories, as shown in the bottom left of the figure.

The complete cp genome of *C. amabile* is 151,532 bp in length with the typical double-stranded circular tetrad structure (Figure 2), containing a small single-copy (SSC) region with a length of 17,366 bp, a large single-copy (LSC) region with a length of 82,902 bp, and a pair of inverted repeats (IRs) regions with a length of 25,632 bp each. The contents of A, T, C, and G are 31.0%, 31.6%, 19.0%, and 18.4%, respectively. The overall GC content of the whole genome is 37.4% (LSC, 35.19%; SSC, 30.86%; IR, 43.09%). In total, 130 genes were annotated, including 85 protein-coding genes (PCGs), eight ribosomal RNA genes (rRNAs), and 37 transfer RNA genes (tRNAs). Of the 130 genes encoded, there are 17 dual-copy genes, including two ribosome macrounit genes (*rpl*2, *rpl*23), one ribosome subunit gene (*rps*7), one NADH dehydrogenase gene (*ndh*B), four rRNA genes (*rrn*5, *rrn*4.5, *rrn*23, *rrn*16), seven tRNA genes (*trn*I-CAU, *trn*I-GAU, *trn*L-CAA, *trn*N-GUU, *trn*R-ACG, *trn*V-GAC, and *trn*A-UGC), and two functionally unknown genes (*ycf*2 and *ycf*1).

### Sequence repeat analysis

A total of 30 SSRs were detected by the online software MISA-web (Table S2), with the numbers of mono-, di-, tri-, tetra-, and pentanucleotides SSRs being 20, 6, 1, 0, and 0, respectively. The number of mononucleotides is the highest, consistent with a previous study in which the mononucleotide had the highest number of occurrences of A and T (Yang et al. 2021). Our result is also consistent with the previous report that the mononucleotide repeats of the cp genome are biased toward A/T repeats (Kuang et al. 2011). Dinucleotide repeats have AT/TA with 4 and 2 occurrences, respectively; trinucleotide has only TTC. Previous studies have suggested that mononucleotides in the cp genome are more likely to exhibit intraspecific variation if located in a noncoding single-copy region (Ebert and Peakall 2009), and analysis of SSRs revealed that 16 mononucleotides were distributed in the noncoding single-copy region. The cp genome of *C. amabile* is enriched in AT, suggesting that it is easier to unpack because AT binding has one less hydrogen bond than GC binding, and the energy required to break AT binding is much lower than that of GC bonds. Therefore, AT repeat motifs are more likely to be included in SSR repeat motifs in the cp genome (Qiao et al. 2019; Zhao et al. 2021).

### Phylogenetic analyses

**Figure 3.**
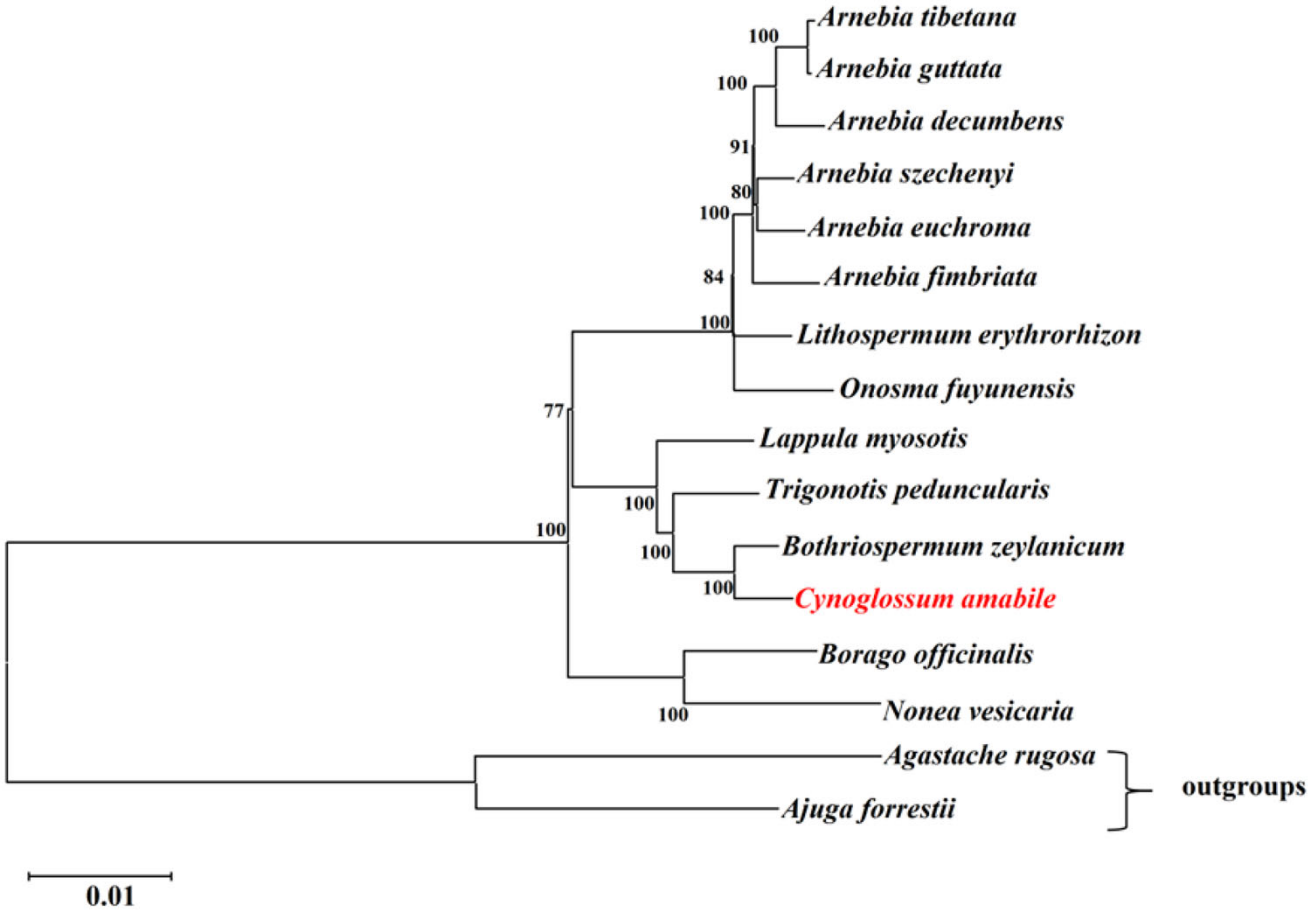
ML phylogenetic tree based on complete chloroplast genome of *C. amabile* and 15 other species. Numbers in the nodes are the bootstrap values from 1000 replicates. The following sequences were used: *Arnebia decumbens* ON529954 (Sun et al. 2022), *Arnebia euchroma* ON529958, *Arnebia fimbriata* ON529943, *Arnebia guttata* ON529956, *Arnebia szechenyi* ON529949, *Arnebia tibetana* MT975392 (Park et al. 2020), *Borago officinalis* NC_046796 (Guo et al. 2020), *Bothriospermum zeylanicum* NC_065834, *Cynoglossum amabile* NC_061706, *Lappula myosotis* NC_060614, *Lithospermum erythrorhizon* MT975394, *Nonea vesicaria* OL335187 (Carvalho Leonardo et al. 2022), *Onosma fuyunensis* NC_049569 (He et al. 2021), *Trigonotis peduncularis* MZ911745 (Wu et al. 2022), *Agastache rugosa* MW760849 (Wang et al. 2021), and *Ajuga forrestii* NC_048512 (Tao et al. 2019).

The phylogenetic analysis revealed that all species of Boraginaceae formed one monophyletic clade. The phylogenetic tree indicated that *Bothriospermum zeylanicum* (J. Jacq.) Druce, 1917 was a sister taxon to *C. amabile* (Figure 3). Our cp genomic data of *C. amabile* contributes to the growing quantity of cp genomes for phylogenetic and evolutionary studies in the family Boraginaceae.

## Conclusions

In this study, the cp genome sequence of *C. amabile* was assembled, annotated, and analyzed. *B. zeylanicum* was found to be closely related to *C. amabile*. These results provide the basis for the molecular identification of *Cynoglossum* plants. This study opens up new avenues for future research and obtains the genomic information of the cps of Boraginaceae taxa on this basis.

## Supplementary Material

Supplemental Material

## Data Availability

The genome sequence data that support the findings of this study are openly available in GenBank of NCBI at https://www.ncbi.nlm.nih.gov under the accession number NC_061706. The associated BioProject, SRA, and Bio-Sample numbers are PRJNA797868, SRR17640219, and SAMN25038855, respectively.
