# Supplementary material for: The complete chloroplast genome of Cynoglossum amabile Stapf & J. R. Drumm., 1906 (Boraginaceae), a traditional Chinese herbal medicine
Source: Mitochondrial DNA B Resour. 2023 Jan 2;8(1):52–6. doi: 10.1080/23802359.2022.2160219 (PMC9815245; doi:10.1080/23802359.2022.2160219)
Supplement: Supplemental Material [file TMDN_A_2160219_SM0056.docx]

**Table and figure captions**

**Table S1.** Details for the chloroplast genome sequences used in the phylogenetic analysis

**Table S2.** SSR information of chloroplast genome of *C. amabile*

**Figure S1.** Overall coverage depth of the chloroplast genome assembly of *C. amabile*

**Figure S2.** Coverage depths of the junctions of the chloroplast genome of *C. amabile*

**Table S1.** Details for the chloroplast genome sequences used in the phylogenetic analysis

| **Family** | **Genus** | **Species** | **GenBank accession number** | **Length** |
| --- | --- | --- | --- | --- |
| [Boraginaceae](https://www.iplant.cn/info/Boraginaceae) | *Arnebia* | *Arnebia decumbens* (Vent.) Coss. et Kral. | ON529954.1 | 149539 bp |
| [Boraginaceae](https://www.iplant.cn/info/Boraginaceae) | *Arnebia* | *Arnebia euchroma* (Royle) Johnst. | ON529958.1 | 150311 bp |
| [Boraginaceae](https://www.iplant.cn/info/Boraginaceae) | *Arnebia* | *Arnebia fimbriata* Maxim. | ON529943.1 | 151977 bp |
| [Boraginaceae](https://www.iplant.cn/info/Boraginaceae) | *Arnebia* | *Arnebia guttata* Bge. | ON529956.1 | 150336 bp |
| [Boraginaceae](https://www.iplant.cn/info/Boraginaceae) | *Arnebia* | *Arnebia szechenyi* Kanitz | ON529949.1 | 151490 bp |
| [Boraginaceae](https://www.iplant.cn/info/Boraginaceae) | *Arnebia* | *Arnebia tibetana* Kurz | MT975392.1 | 150465 bp |
| [Boraginaceae](https://www.iplant.cn/info/Boraginaceae) | *Borago* | *Borago officinalis* L. | NC_046796.1 | 149835 bp |
| [Boraginaceae](https://www.iplant.cn/info/Boraginaceae) | *Borago* | *Bothriospermum* zeylanicum (J. Jacquin) Druce | NC_065834.1 | 152117 bp |
| [Boraginaceae](https://www.iplant.cn/info/Boraginaceae) | *Cynoglossum* | *Cynoglossum amabile* Stapf & J. R. Drumm. | NC_061706.1 | 151532 bp |
| [Boraginaceae](https://www.iplant.cn/info/Boraginaceae) | *Lappula* | *Lappula myosotis* Moench | NC_060614.1 | 146668 bp |
| [Boraginaceae](https://www.iplant.cn/info/Boraginaceae) | *Lithospermum* | *Lithospermum erythrorhizon* Sieb. et Zucc. | MT975394.1 | 149316 bp |
| [Boraginaceae](https://www.iplant.cn/info/Boraginaceae) | *Nonea* | *Nonea vesicaria* (L.) Rchb. | OL335187.1 | 151009 bp |
| [Boraginaceae](https://www.iplant.cn/info/Boraginaceae) | *Onosma* | *Onosma fuyunensis* Y.He & Q.R.Liu | NC_049569.1 | 150612 bp |
| [Boraginaceae](https://www.iplant.cn/info/Boraginaceae) | *Trigonotis* | *Trigonotis peduncularis* (Trev.) Benth. ex Baker et Moore | MZ911745.1 | 147508 bp |
| [Lamiaceae](https://www.iplant.cn/info/Lamiaceae) | *Agastache* | *Agastache rugosa* (Fisch. et Mey.) O. Ktze. | MW760849.1 | 152030 bp |
| [Lamiaceae](https://www.iplant.cn/info/Lamiaceae) | *Ajuga* | *Ajuga forrestii* Diels | NC_048512.1 | 150492 bp |

**Table S2.** SSR information of chloroplast genome of *C. amabile*

| **ID** | **SSR type** | **SSR** | **start** | **end** | **location** | **Region** |
| --- | --- | --- | --- | --- | --- | --- |
| 1 | p1 | (T)11 | 2210 | 2220 | intron(trnK-UUU) | LSC |
| 2 | p2 | (TA)6 | 6333 | 6344 | IGS(rps16, trnQ-UUG) | LSC |
| 3 | p1 | (A)10 | 8231 | 8240 | IGS(psbI, trnS-GCU) | LSC |
| 4 | p1 | (T)10 | 8569 | 8578 | IGS(trnS-GCU, trnG-UCC) | LSC |
| 5 | p1 | (A)11 | 8860 | 8870 | IGS(trnS-GCU, trnG-UCC) | LSC |
| 6 | c | (TA)6ttagtattagtgcattattgaatatacaattcccgaaaatttgtaacatacaatccca(T)12 | 9847 | 9928 | IGS(trnG-UCC, trnR-UCU) | LSC |
| 7 | p1 | (T)10 | 11661 | 11670 | IGS(atpA, atpF) | LSC |
| 8 | p1 | (T)12 | 16338 | 16349 | IGS(rps2, rpoC2) | LSC |
| 9 | p1 | (T)12 | 18558 | 18569 | rpoC2 | LSC |
| 10 | p2 | (AT)5 | 19939 | 19948 | rpoC2 | LSC |
| 11 | p2 | (AT)5 | 26664 | 26673 | rpoB | LSC |
| 12 | p2 | (TA)5 | 31856 | 31865 | IGS(trnT-GGU, psbD) | LSC |
| 13 | p3 | (TTC)4 | 34863 | 34874 | psbC | LSC |
| 14 | p1 | (C)10 | 39930 | 39939 | psaA | LSC |
| 15 | p1 | (A)10 | 44069 | 44078 | intron(ycf3) | LSC |
| 16 | p1 | (A)12 | 44370 | 44381 | IGS(ycf3, trnS-GGA) | LSC |
| 17 | p2 | (AT)6 | 45054 | 45065 | IGS(ycf3, trnS-GGA) | LSC |
| 18 | p1 | (T)10 | 46427 | 46436 | IGS(rps4, trnT-UGU) | LSC |
| 19 | p1 | (T)10 | 50496 | 50505 | IGS(ndhC, trnV-UAC) | LSC |
| 20 | p1 | (T)10 | 57855 | 57864 | IGS(accD, psaI) | LSC |
| 21 | p1 | (T)10 | 59557 | 59566 | IGS(ycf4, cemA) | LSC |
| 22 | p2 | (AT)5 | 65633 | 65642 | IGS(trnP-UGG, psaJ) | LSC |
| 23 | p1 | (A)10 | 69385 | 69394 | intron(clpP) | LSC |
| 24 | p1 | (A)10 | 69863 | 69872 | intron(clpP) | LSC |
| 25 | p1 | (A)10 | 111343 | 111352 | IGS(ndhF, rpl32) | SSC |
| 26 | p1 | (A)11 | 115171 | 115181 | IGS(ndhD, psaC) | SSC |
| 27 | c | (A)11gaattagacacgatattagtgttcataaatcatgacaacaatctctttaaatacaaatatgtatccaggaaaaaaaaagatttc(T)12 | 118579 | 118685 | intron(ndhA) | SSC |
| 28 | p1 | (T)10 | 123223 | 123232 | ycf1 | SSC |
| 29 | p1 | (T)11 | 123382 | 123392 | ycf1 | SSC |
| 30 | c | (T)10ctcttttgtaattctttctatttgatttcgaattatattgattctattagtcagatctttca(T)11 | 124064 | 124146 | ycf1 | SSC |

*
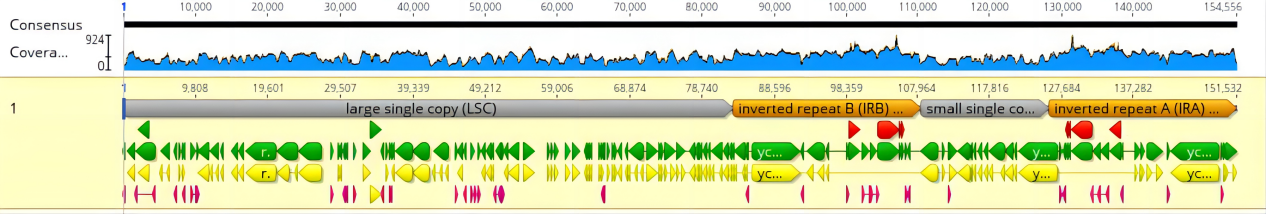
*

**Figure S1.** Overall coverage depth of the chloroplast genome assembly of *C. amabile*. This figure was generated using Geneious Prime by aligning DNA-Seq data to the complete cp genome). The upper area showed the coverage depth of each locus of the genome. The lower area showed the length, gene information and tetrad region information of the genome. Through the figure, each locus of the genome can be covered by reads. It can be used as evidence that the genome is circular.

*
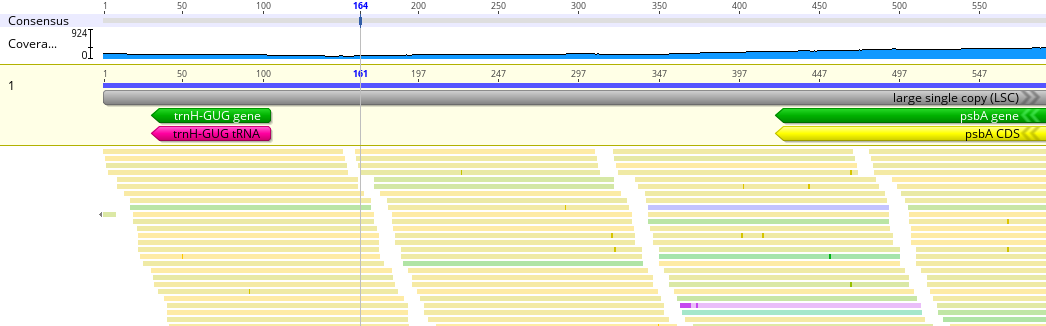
*

*
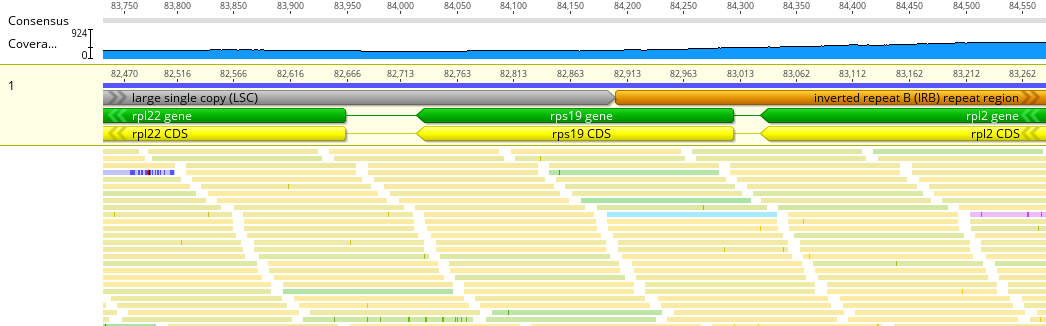
*

*
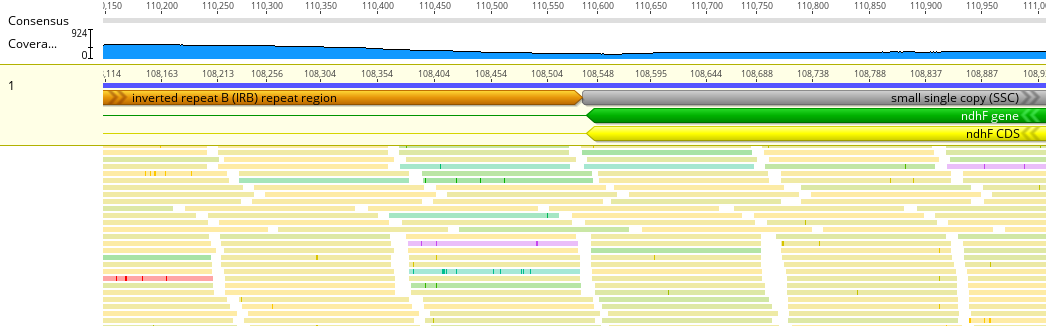
*

*
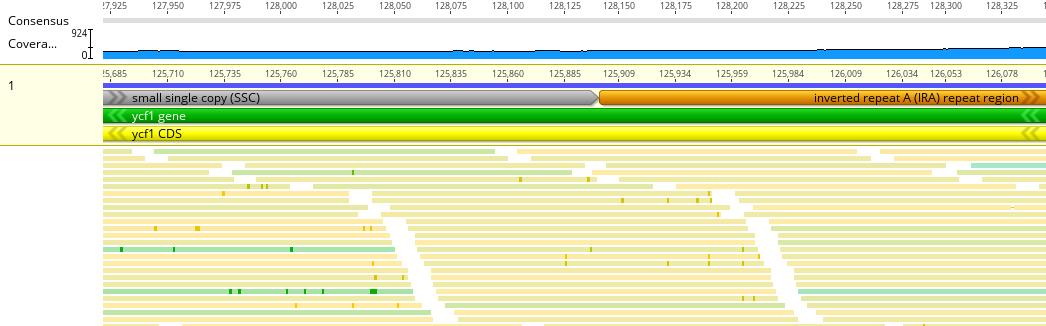
*

*
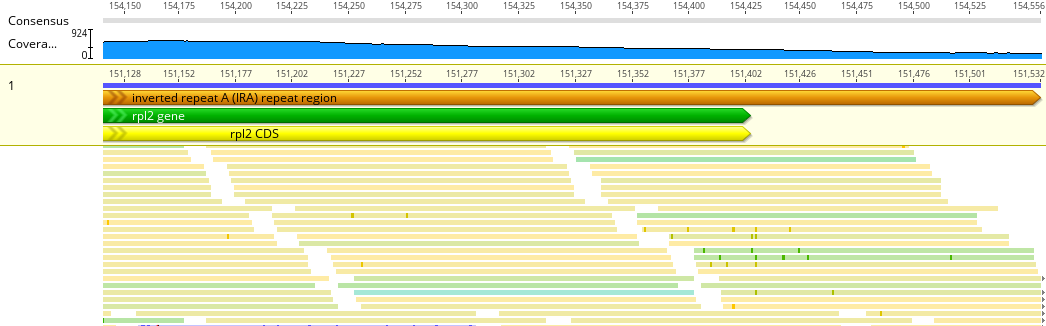
*

**Fig S2.** Coverage depths of the junctions of the chloroplast genome of *C. amabile*. These figures were generated using Geneious Prime by aligning DNA-Seq data to the complete cp genome. In these figures, the upper area showed the coverage depth of the junctions of the genome. The middle area showed the junctions of the genome. The lower area showed the reads used for alignment. These figures can be used as evidence that there is no problem with assembly of the junctions of the chloroplast genome.
